# Using Paper Texture for Choosing a Suitable Algorithm for Scanned Document Image Binarization

**DOI:** 10.3390/jimaging8100272

**Published:** 2022-10-05

**Authors:** Rafael Dueire Lins, Rodrigo Bernardino, Ricardo da Silva Barboza, Raimundo Correa De Oliveira

**Affiliations:** 1 Centro de Informática, Universidade Federal de Pernambuco, Recife 50670-901, PE, Brazil; 2 Departamento de Computação, Universidade Federal Rural de Pernambuco, Recife 55815-060, PE, Brazil; 3 Coordenação de Engenharia da Computação, Escola Superior de Tecnologia, Universidade do Estado do Amazonas, Manaus 69410-000, AM, Brazil

**Keywords:** document binarization, historical documents, DIB dataset, scanned documents, binarization competitions, binarization algorithms

## Abstract

The intrinsic features of documents, such as paper color, texture, aging, translucency, the kind of printing, typing or handwriting, etc., are important with regard to how to process and enhance their image. Image binarization is the process of producing a monochromatic image having its color version as input. It is a key step in the document processing pipeline. The recent Quality-Time Binarization Competitions for documents have shown that no binarization algorithm is good for any kind of document image. This paper uses a sample of the texture of the scanned historical documents as the main document feature to select which of the 63 widely used algorithms, using five different versions of the input images, totaling 315 document image-binarization schemes, provides a reasonable quality-time trade-off.

## 1. Introduction

The process of converting a color image into its black-and-white (or monochromatic) version is called binarization or thresholding. The binary version of document images are, in general, more readable by humans, and save storage space [1,2] and communication bandwidth in networks, as the size of binary images is often orders of magnitudes smaller than the original gray or color images; they also use less toner for printing. Thresholding is a key preprocessing step for document transcription via OCR, which allows document classification and indexing.

No single binarization algorithm is good for all kinds of document images, as is demonstrated by the recent Quality-Time Binarization Competitions [3,4,5,6,7]. The quality of the resulting image depends on a wide variety of factors, from the digitalization device and its setup to the intrinsic features of the document, from the paper color and texture to the way the document was handwritten or printed. The time elapsed in binarization also depends on the document features and varies widely between algorithms. A fundamental question arises here: if the document features are deterministic for the quality output of the binary image and there is also a large time-performance variation, and there is a growing number of binarization algorithms, how does one choose an algorithm that provides the best quality-time trade-off? Most users tend to binarize a document image with one of the classical algorithms, such as Otsu [8] or Sauvola [9]. Often, the quality of the result is not satisfactory, forcing the user to enhance the image through filtering (salt-and-pepper, etc.) or to hand-correct the image.

The case of the binarization of photographed documents is even more complex than scanned ones, as the document image has uneven resolution and illumination. The ACM DocEng Quality-Time Binarization Competitions for Photographed Documents [5,6,7] have shown that in addition to the physical document characteristics, the camera features and its setup (whether the in-built strobe flash is on or off) influence which binarization algorithm performs the best in quality and time performance. The recent paper [10] presents a new methodology to choose the “best” binarization algorithm in quality and time performance for documents photographed with portable digital cameras embedded in cell phones. It assesses 61 binarization algorithms to point out which binarization algorithm quality-time performs the best for OCR preprocessing or image visualization/printing/network transmission for each of the tested devices and setup. It also chooses the “overall winner”, and the binarization algorithms that would be the “first-choice” in the case of a general embedded application, for instance.

The binarization of scanned documents is also a challenging task. The quality of the resulting image varies not only with the set resolution of the scanner (today, the “standard” is either 200 or 300 dpi), but it also depends heavily on the features of each document, such as paper color and texture, how the document was handwritten or printed, the existence of physical noises [11], etc. Thus, it is important to have some criteria to point out which binarization algorithm, among the best algorithms today, provides the best quality-time trade-off for scanned documents.

Traditionally, binarization algorithms convert the color image into gray-scale before performing binarization. Reference [12] shows that the performance of binarization algorithms may differ if the algorithm is fed with the color image, its gray-scale converted image or one of its R, G, or B channels. Several authors [13,14] show that texture analysis plays an important role in document image processing. Two of the authors of this paper showed that the analysis of paper texture allows one to determine the age of documents for forensic purposes [15], avoiding document forgeries. This paper shows that by extracting a sample of the paper (background) texture of a scanned document, one can have a good indication of one of the 315 binarization schemes tested [12] that provides a suitable quality monochromatic image, with a reasonable processing time to be integrated into a document processing pipeline.

## 2. Materials and Methods

This work made use of the International Association for Pattern Recognition (IAPR) document image binarization (DIB) platform (https://dib.cin.ufpe.br, last accessed on 24 August 2022), which focuses on document binarization. It encompasses several datasets of document images of historical, bureaucratic, and ordinary documents, which were handwritten, machine-typed, offset, laser- and ink-jet printed, and both scanned and photographed; several documents had corresponding ground-truth images. In additon to being a document repository, the DIB platform encompasses a synthetic document image generator, which allows the user to create over 5.5 million documents with different features. As already mentioned, Ref. [12] shows that binarization algorithms, in general, yield different quality images whenever fed with the color, gray-scale-converted, and R, G, and B channels. Here, 63 classical and recently published binarization algorithms are fed with the five versions of the input image, totaling 315 different binarization schemes. The full list of the algorithms used is presented in Table 1 and Table 2, along with a short description and the approach followed in each of them.

Ref. [63] presents a machine learning approach for choosing among five binarization algorithms to binarize parts of a document image. Another interesting approach to enhance document image binarization is proposed in [64] and consists of analyzing the features of the original document to compose the result of the binarization of several algorithms to generate a better monochromatic image. Such a scheme was tested with 25 binarization algorithms, and it performed more than 3% better than the first rank in the H-DIBCO 2012 contest in terms of F-measure. The time-processing cost of such a scheme is prohibitive if one considers processing document batches, however.

**Figure 1:**
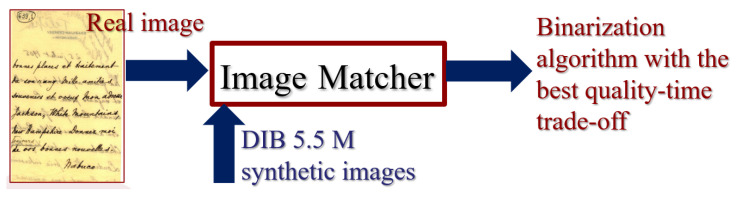
DIB image matcher.

One of the aims raised by the researchers in the DIB platform team is to develop an “image matcher” in such a way that given a real-world document, it looks for the synthetic document that better matches its features, as sketched in Figure 1. For each of the 5.5 million synthetic documents in the DIB platform, one would have the algorithms that would yield the best quality-time performance for document readability or OCR transcription. Thus, the match of the “real-world” document and the synthetic one would point out which binarization algorithm would yield the “best” quality-time performance for the real-world document. It is fundamental that the Image Matcher is a very lightweight process not to overload the binarization processing time. If one or a small set of document features provide enough information to make such a good choice, it is more likely that it will be for the image-matcher to be fast enough to be part of a document processing pipeline.

**Figure 2:**
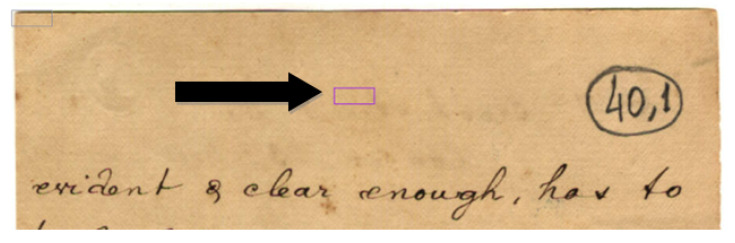
DIB—Choosing a texture pattern.

In this paper, the image texture is taken as a key for selecting the real-world image that more closely resembles another real-world document for which one has a ground-truth monochromatic image of reference. Such images were carefully chosen from the set of historical documents in the DIB platform such as to match a large number of historical documents of interest from the late 19th century to today. To extract a sample of the texture, one manually selects a window of 120 × 60 pixels from the document to be binarized, as shown in Figure 2. Only one window from each image was cropped in such a way that there was no presence of text from the front or any back-to-front interference. A vector of features is built, taking into account each RGB channel of the sample, the image average filtered (R + G + B)/3, and its gray-scale equivalent. Seven statistical measures are taken and placed in a vector: mean, standard deviation, mode, minimum value, maximum value, median, and kurtosis. This results in a vector containing 28 features, which describes the overall color and texture characteristics.

**Figure 3:**
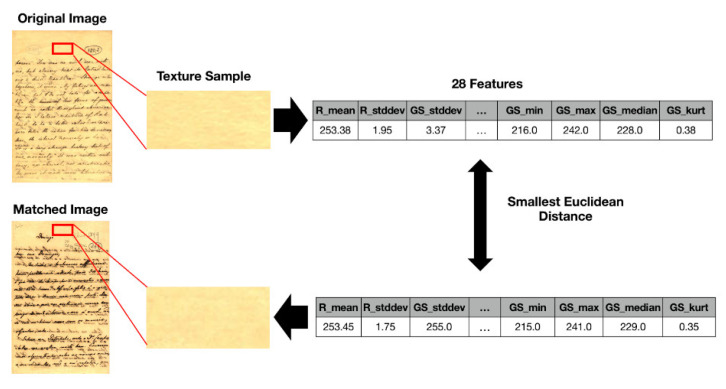
Example of matching real-world images by texture.

In this study, 40 real-world images are used, and the Euclidean distance between the texture vectors is used to find the 20 pairs of most similar documents. The texture with the smallest distance is chosen, and its source document image is used to determine the best binarization algorithm. Figure 3 illustrates how such a process is applied to a sample image and the chosen texture.

## 3. Binarization Algorithm Selection Based on the Paper Texture

**Figure 4:**
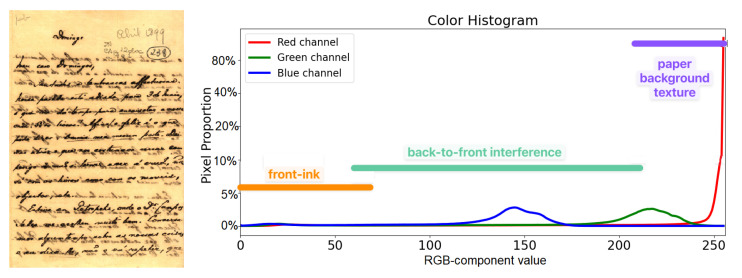
Pixel color distributions in a document image with strong back-to-front interference.

In a real-world document, one expects to find three overlapping color distributions. This includes one that corresponds to the plain paper background, which becomes the paper texture, which should yield white pixels in the monochromatic image. The second distribution tends to be a much narrower Gaussian that corresponds to the printing or writing, which is mapped onto black pixels in the binary image. The third distribution, the back-to-front interference [11,65] overlaps the other two distributions, bringing one of the most important causes of binarization errors. Figure 4 presents a saple image with the corresponding color distributions.

Deciding which binarization algorithm to use in a document tends to be a “wild guess”, a user-experience-based guess, or an a posteriori decision, which means one uses several binarization algorithms and chooses the image that “looks best” as a result. Binarization time is seldom considered. One must agree that the larger the number of binarization algorithms one has, the harder it is to guess the ones that will perform well for a given document. Ideally, the Image Matcher under development in the DIB-platform would estimate all the image parameters (texture type, kind of writing or printing, the color of ink, intensity of the back-to-front interference, etc.) to pinpoint which of the over 5.5 million synthetic images best matches the features of the “real world” document to be binarized. If that synthetic image is known, one would know which of the 315 binarization schemes assessed here would offer the best quality-time balance for that synthetic image.

This paper assumes that by comparing the paper texture between two real-world documents, one of which knows which binarization algorithm presents the best quality-time trade-off, one can use that algorithm on the other document, yielding acceptable quality results. Cohen’s Kappa [66,67] (denoted by *k*) is used here as a quality measure:

(1) $$k=\frac{P_{O}-P_{C}}{1-P_{C}},$$

which compares the observed accuracy with an expected accuracy, assessing the classifier performance. $P_{O}$ is the number of correctly mapped pixels (accuracy) and $P_{C}$ is

(2) $$P_{C}=\frac{n_{bf}\times n_{gf}+n_{bb}\times n_{gb}}{N^{2}},$$

where $n_{bf}$ and $n_{bb}$ are the number of pixels mapped as foreground and background on the binary image, respectively, and $n_{gf}$ and $n_{gb}$ are the number of foreground and background pixels on the GT image, and *N* is the total number of pixels. The ranking for the pixels is defined by sorting the measured kappa in ascending order.

The peak signal-noise ratio (PSNR), distance reciprocal distortion (DRD) and F-Measure (FM) have been used for a long time to assess binarization results [68,69], becoming the chosen measures for nearly all studies in this area. Thus, they are also provided, even though the ranking process only takes Cohen’s Kappa into account. The PSNR for a M×N image is defined as the peak signal power to average noise power, which, for 8-bit images, is

(3) $$PSNR=10\log_{10}\frac{255^{2}\cdot MN}{\sum_{i}\sum_{j}{\left(x\left(i,j\right)-y\left(i,j\right)\right)}^{2}}.$$

The DRD [70] correlates the human visual perception with the quality of the generated binary image. It is computed by

(4) $$DRD=\frac{1}{NUBN(GT)}\sum_{k=1}^{S}DRD_{ij}\left|B\left(i,j\right)-GT\left(i,j\right)\right|$$

(5) $$DRD_{ij}=\sum_{x=-2}^{2}\sum_{y=-2}^{2}W_{xy}\left|B\left(i+x,j+y\right)-G\left(i+x,j+y\right)\right|,$$

where NUBN (GT) is the number of non-uniform 8×8 binary blocks in the ground-truth (GT) image, *S* is the flipped pixels and $DRD_{ij}$ is the distortion of the pixel at position (i,j) in relation to the binary image (B), which is calculated by using a 5 × 5 normalized weight matrix $W_{xy}$ as defined in [70]. $DRD_{ij}$ equals to the weighted sum of the pixels in the 5 × 5 block of the GT that differ from the centered kth flipped pixel at (x,y) in the binarization result image *B*. The smaller the DRD, the better.

The F-Measure is computed as

(6) $$FM=\frac{2\times Recall\times Precision}{Recall+Precision},$$

where $Recall=\frac{TP}{TP+FN}$, $Precision=\frac{TP}{TP+FP}$ and TP, FP, FN denote the true positive, false positive and false negative values, respectively.

Once the matching image (the most similar) is found, the best quality-time algorithm is used to binarize the original image. Algorithms with the same kappa are in the same ranking position. Several algorithms have a similar processing time. Among the top-10 in terms of quality, the fastest is chosen as the best quality-time binarization algorithm. This paper conjectures that considering two documents that were similarly printed (handwritten, offset printed, etc.) and have similar textures, if the best quality-time algorithm is known for one image, that same algorithm could be applied to the other image, yielding high-quality results. No doubt that if a larger number of document features besides the document texture, such as the strength of the back-to-front interference, the ink color and kind of pen, the printing method, etc. were used, the chances of selecting the best quality binarization scheme would be larger, but could imply in a prohibitive time overhead. It is also important to stress that the number of documents with back-to-front interference is small in most document files, and the ones with strong interference is even smaller. In the case of the bequest [71] of Joaquim Nabuco (1849/1910, Brazilian statesman and writer and the first Brazilian ambassador to the U.S.A.), for instance, the number of letters is approximately 6500, totaling about 22,000 pages. Only 180 documents were written on both sides in translucent paper, of which less than 10% of them exhibit strong back-to-front interference. Even in those documents, the paper texture plays an important role in the parameters of the binarization algorithms. Thus, in this paper, one assumes that the paper texture is the key information for choosing a suitable binarization scheme that has a large probability of being part of an automatic document processing pipeline. Evidence that such a hypothesis is valid is shown in the next section.

## 4. Results

In order to evaluate the automatic algorithm selection based on the texture, 26 handwritten and 14 typewritten documents were carefully selected from the DIB platform such that they are representative of a large number of real-world historical documents. Such documents belong to the Nabuco bequest [71] and were scanned in 200 dpi. Table 3 presents the full size of each document used in this study. All of them have a ground-truth binary image. The Euclidean distance between the feature vector of their paper textures was used to find the pairs of most similar documents. Five versions of the original and matched image were used in the final ranking.

The results and the images are described in Table 4, Table 5, Table 6, Table 7, Table 8, Table 9, Table 10, Table 11 and Table 12. The letter that follows the algorithm name indicates the version of the input document image used, that as shown in [12] yields monochromatic images of different quality with different processing times:

- **C:** all RGB channels (color)
- **R:** the red channel
- **G:** the green channel
- **B:** the blue channel
- **L:** luminance image, calculated as 0.299∗R+0.587∗G+0.114∗B

The other parts stand for:

1. **Original Image:** the image one wants to binarize.
2. **Matched Image:** the image which one already has the algorithm that yields the best quality-time trade-off amongst all the 315 binarization schemes.
3. **Textures samples:** sample of the paper background of the original image (left) used to select the texture matched image (right), whose sample is presented below each document
4. **Results Table:** the best 10 algorithms for the original image
5. **Direct Binarization:** the best quality-time algorithm and corresponding binary image according to the ranking of all 315 binarization schemes. The choice is made by directly looking at the results of all algorithms.
6. **Texture-based Binarization:** the best quality-time algorithm of the matched image and the corresponding monochromatic version of the original image binarized with the chosen algorithm.

**Figure 5:**
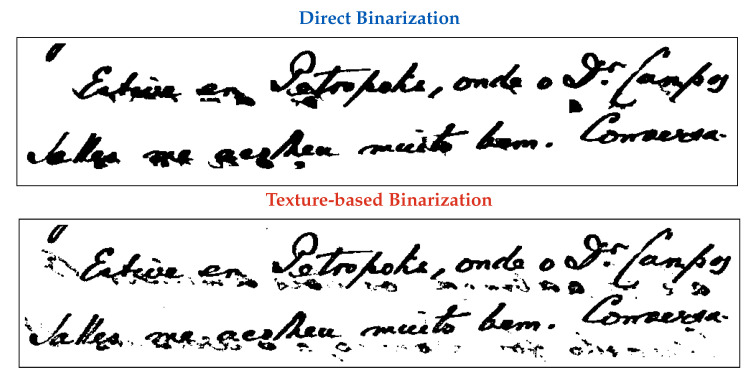
Zoom in a part of a document image HW 05 with strong back-to-front interference binarized using the direct (Jia-Shi [38]) and texture-based (Wolf [72]) methods.

The algorithm choice was appropriate for all the presented images, as can be noted by visually inspecting the binary images, their quality ranking, and the kappa, PSNR, DRD, and F-Measure values. For Table 4, Table 6, Table 7, and Table 9, the selected algorithm was at rank 5 or more and did not yield a significantly worse image in those cases. The difference in kappa was smaller than 10%, except in the case of the image shown in Table 8, in which the kappa reached 12%. It is interesting to observe that for the image shown in Table 8, an image with strong back-to-front interference, although the value of kappa has the highest percent difference of all the tested images, the monochromatic image produced by using the texture binarization scheme proposed here is visually more pleasant and readable for humans than the scheme that yields the best kappa, as may be observed in the zoomed image shown in Figure 5. One may see that the texture-based choice of the binarization scheme leaves some noise in areas that correspond to the back-to-front interference, most of which could be removed with a salt-and-pepper filter. As previously remarked here, images with strong back-to-front interference tend to be rare in any historical document file.

In the case of the document image HW 04, presented in Table 7, although the difference in kappa is 7.3% , visually inspecting the resulting binary image, it is really close in quality to the actual best in terms of quality, which implies the choice based on texture does indicate a good option of binarization algorithm even with a relatively lower rank, although the Howe algorithm [32] used to binarize the matched image HW 09, has a much higher processing time than the da Silva–Lins–Rocha algorithm [2] (dSLR-C), the top quality algorithm using direct binarization.

It is also relevant to say that there is a small degree of subjectivity in the whole process as the ground-truth images of historic documents are hand-processed. If one looks at Table 10, one may also find some differences in the produced images that illustrate such subjectivity. The result of the direct binarization using Li–Tam algorithm [41] yields an image with a high kappa of 0.94 with much thicker strokes than the one chosen by the texture-based method, the Su–Lu algorithm [57], both of which were fed with the gray-scale image obtained by using the conventional luminance equation. Although the kappa of the Su–Lu binarized image is 0.89, the resulting image is as readable as the Li–Tam one, a phenomenon which is somehow similar to the one presented in Figure 5. The main idea of the proposed methodology is not to find exactly the same best quality-time algorithm as directly binarizing, but one algorithm that yields satisfactory results.

## 5. Conclusions

Document binarization is a key step in many document processing pipelines; thus it is important to be performed quickly and with high quality. Depending on the intrinsic features of the scanned document image, the quality-time performance of the binarization algorithms known today varies widely. The search for a document feature that is possible to be extracted automatically with a low time complexity that may provide an indication of which binarization algorithm provides the best quality-time trade-off is thus of strategic importance. This paper takes the document texture as such a feature.

The results presented have shown that the document texture information may be satisfactorily used as a way to choose which binarization algorithm to apply to scanned historical documents, and how the input image should be if the original color image, its gray-scale conversion or one of its RGB channels is to be successfully scanned. The choice of the algorithms is based on the use of real images that “resemble” the paper background of the document to be binarized. A sample of the texture of the document is collected and compared with the remaining 39 different paper textures used for handwritten or machine typed documents, each of which points to an algorithm that provides the best quality-time trade-off for the synthetic document. The use of that algorithm in the real-world document to be binarized was assessed here and yielded results that may be considered of good quality and quickly produced, both for image readability by humans or automatic OCR transcription.

This paper presents evidences that by matching the textures of scanned documents, one can find suitable binarization algorithms for a given new image. The methodology presented may be enhanced further by including new textures and binarization schemes. The inclusion of new textures may narrow the euclidean distance between the image to be binarized document and the existing textures in the dataset. The choice of the most suitable binarization scheme for the document with the new texture may be done by the visual inspection of the result of the top-ranked binarization algorithms of the document image with the closest Euclidean distance of the images already in the reference dataset.

A number of issues remain open for further work, however. The first one is automating the process of texture sampling and matching in such a way as not to be a high overload on the binarization process as a whole. This may also involve the collection of texture samples in different parts of the document to avoid collecting parts either printed with back-to-front interference or other physical noises, such as stains or holes. The second point is trying to minimize the number of features in the vector-of-features to be matched with the vector-of-features of the synthetic textures. The third point is attempting to find a better matching strategy than simply calculating the Euclidean distance between the vectors, as done here, perhaps by using some kind of clustering.

## Figures and Tables

**Table 1:** Tested binarization algorithms—Part 1.

Method	Category	Description
Akbari_1 [16]	Deep Learning	Segnet network architecture fed by multichannel images (wavelet sub bands)
Akbari_2 [16]	Deep Learning	Variation of Akibari_1 with multiple networks
Akbari_3 [16]	Deep Learning	Variation of Akibari_1 where fewer channels are used
Bataineh [17]	Local threshold	based on local and global statistics
Bernsen [18]	Local threshold	Uses local image contrast to choose threshold
Bradley [19]	Local threshold	Adaptive thresholding using the integral image of the input
Calvo-Zaragoza [20]	Deep learning	Fully convolutional Encoder–decoder FCN with residual blocks
CLD [21]	Local threshold	Contrast enhancement followed by adaptive thresholding and artifact removal
CNW [22]	Local threshold	Combination of Niblack and Wolf’s algorithm
dSLR [23]	Global threshold	Uses Shannon entropy to find a global threshold
DeepOtsu (SL) [24]	Deep Learning	Neural networks learn degradations and global Otsu generates binarization map
DE-GAN [25]	Deep Learning	Uses a conditional generative adversarial network
DiegoPavan (DP) [4]	Deep Learning	Downscale image to feed a DE-GAN network
DilatedUNet [5]	Deep Learning	Downsample to smooth image and use a dilated convolutional layer to correct the feature map spatial resolution
DocDLinkNet [26]	Deep Learning	D-LinkNet architecture with document image patches
DocUNet (WX) [3]	Deep Learning	A hybrid pyramid U-Net convolutional network is fed with morphological bottom-hat transform enhanced document images
ElisaTV [27]	Local threshold	Background estimation and subtraction
Ergina-Global [28]	Global threshold	Average color value and histogram equalization
Ergina-Local [29]	Local threshold	Detects where to apply local thresholding after a applying a global one
Gattal [30]	Clustering	Automatic Parameter Tuning of K-Means Algorithm
Gosh [31]	Clustering	Clustering applied to a superset of foreground estimated by Niblack’s algorithm
Howe [32]	CRF Laplacian	unary term and pairwise Canny-based term
Huang [33]	Global threshold	Minimizes the measures of fuzzines
HuangBCD (AH${}_{1}$) [4]	Deep Learning	BCD-Unet based model binarize and combine image patches
HuangUNet (AH${}_{2}$) [4]	Deep Learning	Unet based model binarize and combine image patches
iNICK [34]	Local threshold	Adaptively sets k in NICK based on global std. dev.
Intermodes [35]	Global threshold	Smooth histogram until only two local maxima
ISauvola [36]	Local threshold	Uses image contrast in combination with Sauvola’s binarization
IsoData [37]	Global threshold	IsoData clulstering algorithm applied to image histogram
Jia-Shi [38]	Edge based	Detecting symmetry of stroke edges
Johannsen-Bille [39]	Global threshold	Minimizes formula based on the image entropy
Kapur-SW [40]	Global threshold	Maximizes formula based on the image entropy
Li-Tam [41]	Global threshold	Minimum cross entropy
Lu-Su [42]	Edge based	Local thresholding near edges after background removal
Mean [43]	Global threshold	Mean of the grayscale levels
Mello-Lins [44]	Global threshold	Uses Shannon Entropy to determine the global threshold. Possibly the first to properly handle back-to-front interference

**Table 2:** Tested binarization algorithms—Part 2.

Method	Category	Description
Michalak [45]	Image Processing	Downsample image to remove low-frequency information and apply Otsu
MO${}_{1}$ [45]	Image Processing	Downsample image to remove low-frequency information and apply Otsu
MO${}_{2}$ [46]	Image Processing	Equalize illumination and contrast, apply morphological dilatation and Bradley’s method
MO${}_{3}$ [47]	Local threshold	Average brightness corrected by two parameters to apply local threshold
MinError [48]	Global threshold	Minimum error threshold
Minimum [35]	Global threshold	Variation of Intermodes algorithm
Moments [49]	Global threshold	Aims to preserve the moment of the input picture
Niblack [50]	Local threshold	Based on window mean and std. dev.
Nick [51]	Local threshold	Adapts Niblack based on global mean
Otsu [8]	Global threshold	Maximize between-cluster variance of pixel intensity
Percentile [52]	Global threshold	Based on partial sums of the histogram levels
Pun [53]	Global threshold	Defines an anisotropy coefficient related to the asymmetry of the histogram
RenyEntropy [54]	Global threshold	Uses Renyi’s entropy similarly as Kapur’s method
Sauvola [9]	Local threshold	Improvement on Niblack
Shanbhag [55]	Global threshold	Improves Kapur’s method; view the two pixel classes as fuzzy sets
Singh [56]	Global threshold	Uses integral sum image prior to local mean calculation
Su-Lu [57]	Edge based	Canny edges using local contrast
Triangle [58]	Global threshold	Based on most and least frequent gray level
Vahid (RNB) [4]	Deep Learning	A pixel-wise segmentation model based on Resnet50-Unet
WAN [59]	Global threshold	Improves Sauvola’s method by shifting up the threshold
Wolf [60]	Local threshold	Improvement on Sauvola with global normalization
Wu-Lu [61]	Global threshold	Minimizes the difference between the entropy of the object and the background
Yen [62]	Global threshold	Multilevel threshold based on maximum correlation criterion
YinYang [5]	Image Processing	Detect background with median of small overllaping windows, extract it and apply Otsu
YinYang21 (JB) [5]	Image Processing	A faster and more effective version of YinYang algorithm
Yuleny [3]	Shallow ML	A XGBoost classifier is trained with features generated from Otsu, Niblack, Sauvola, Su and Howe algorithms

**Table 3:** Size of the test images in pixels.

Image	Size	Image	Size	Image	Size	Image	Size
HW01	888 × 1361	HW11	907 × 1383	HW21	1077 × 1345	TW05	1602 × 2035
HW02	915 × 1358	HW12	937 × 1372	HW22	894 × 1387	TW06	1551 × 1947
HW03	920 × 1374	HW13	924 × 1381	HW23	925 × 1376	TW07	1212 × 1692
HW04	911 × 1426	HW14	895 × 1373	HW24	992 × 1552	TW07	1212 × 1692
HW05	1021 × 1586	HW15	999 × 1557	HW25	912 × 1375	TW09	1619 × 1961
HW06	1024 × 1550	HW16	890 × 1380	HW26	891 × 1381	TW10	1599 × 2067
HW07	898 × 1389	HW17	954 × 1401	TW01	1645 × 2140	TW11	1701 × 1957
HW08	1016 × 1570	HW18	1049 × 1670	TW02	1660 × 2186	TW12	1677 × 2179
HW09	866 × 1354	HW19	917 × 1372	TW03	1581 × 2119	TW13	1692 × 2193
HW10	1021 × 1579	HW20	1050 × 1326	TW04	1575 × 1989	TW14	1671 × 2165

**Table 4:** Results for image matching with image HW 01.

Binarization Results							Original Image	Matched Image
for the Original Image							HW 01	HW 12
#	**Algorithm**	**Kappa**	**PSNR**	**DRD**	**FM**	**Time**	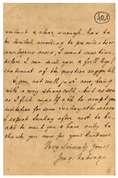	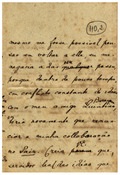
1	IsoData-C	0.92	20.07	1.50	92.11	0.01		
1	IsoData-L	0.92	20.06	1.50	92.06	0.01		
1	Otsu-C	0.92	20.10	1.48	92.15	0.00		
1	Otsu-L	0.92	20.06	1.50	92.06	0.00		
1	Gattal-C	0.92	20.12	1.46	92.13	45.59		
1	Gattal-L	0.92	20.06	1.50	92.06	45.87		
2	dSLR-C	0.91	19.67	1.69	91.54	0.02		
2	dSLR-G	0.91	19.82	1.62	91.69	0.02		
...	...	...	...	...	...	...		
2	MO${}_{1}$-R	0.91	19.81	1.55	91.58	0.14		
**Original Texture**			**Matched Texture**				Direct Binarization	Texture-based
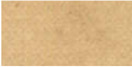			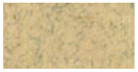				Otsu-C 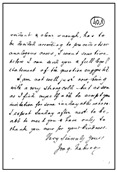	MO_1_-R 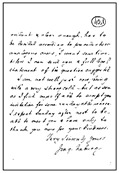
**Direct Binarization**								
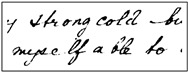								
**Texture-based binarization**								
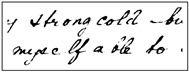								

**Table 5:** Results for image matching with image HW 02.

Binarization Results							Original Image	Matched Image
for the Original Image							HW 02	HW 16
#	**Algorithm**	**Kappa**	**PSNR**	**DRD**	**FM**	**Time**	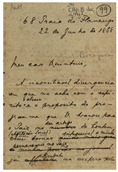	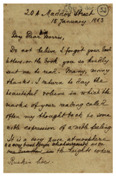
1	Li-Tam-C	1.00	34.52	0.11	99.73	0.01		
2	dSLR-G	0.99	28.01	0.31	98.80	0.01		
2	dSLR-L	0.99	28.39	0.29	98.90	0.01		
2	Intermodes-G	0.99	29.07	0.27	99.07	0.01		
2	Intermodes-L	0.99	27.78	0.34	98.76	0.01		
2	Li-Tam-G	0.99	29.07	0.27	99.07	0.01		
2	Li-Tam-L	0.99	29.77	0.24	99.21	0.01		
3	dSLR-R	0.98	26.90	0.38	98.45	0.01		
...	...	...	...	...	...	...		
8	dSLR-C	0.93	20.56	1.73	93.77	0.01		
**Original Texture**			**Matched Texture**				Direct Binarization	Texture-based
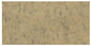			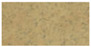				Li-Tam-C 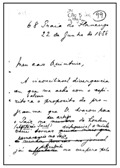	dSLR-C 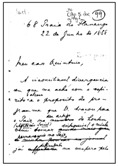
**Direct Binarization**								
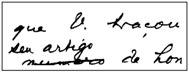								
**Texture-based binarization**								
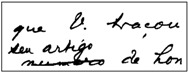								

**Table 6:** Results for image matching with image HW 03.

Binarization Results							Original Image	Matched Image
for the Original Image							HW 03	HW 12
#	**Algorithm**	**Kappa**	**PSNR**	**DRD**	**FM**	**Time**	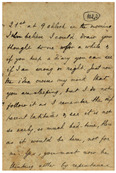	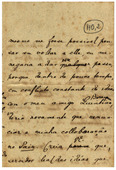
1	ElisaTV-G	0.96	22.52	0.91	95.83	1.60		
1	ElisaTV-L	0.96	22.52	0.91	95.85	1.59		
1	MO${}_{1}$-C	0.96	23.24	0.82	96.51	0.01		
1	MO${}_{1}$-G	0.96	22.91	0.92	96.24	0.01		
1	MO${}_{1}$-L	0.96	23.15	0.85	96.43	0.01		
1	MO${}_{1}$-R	0.96	22.87	0.86	96.19	0.01		
2	dSLR-G	0.95	22.00	1.07	95.21	0.01		
2	dSLR-L	0.95	22.10	1.03	95.31	0.01		
2	dSLR-R	0.95	22.00	1.02	95.30	0.01		
2	Huang-R	0.95	21.96	1.03	95.29	0.01		
**Original Texture**			**Matched Texture**				Direct Binarization	Texture-based
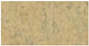			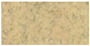				MO${}_{1}$-C 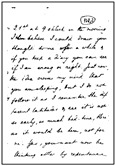	MO${}_{1}$-R 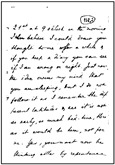
**Direct Binarization**								
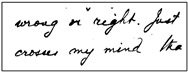								
**Texture-based binarization**								
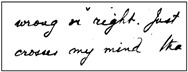								

**Table 7:** Results for image matching with image HW 04.

Binarization Results							Original Image	Matched Image
for the Original Image							HW 04	HW 09
#	**Algorithm**	**Kappa**	**PSNR**	**DRD**	**FM**	**Time**	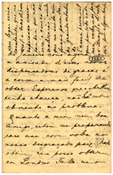	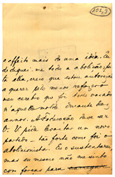
1	dSLR-C	0.96	22.26	4.15	96.70	0.01		
1	Intermodes-C	0.96	22.08	4.28	96.53	0.01		
1	Intermodes-G	0.96	22.01	4.36	96.48	0.01		
1	Intermodes-L	0.96	22.15	4.22	96.60	0.01		
1	Intermodes-R	0.96	21.62	4.68	96.17	0.01		
1	Li-Tam-L	0.96	21.68	4.64	96.17	0.01		
1	Sauvola-C	0.96	21.69	4.68	96.13	0.03		
1	Sauvola-G	0.96	21.87	4.52	96.30	0.03		
...	...	...	...	...	...	...		
8	Howe-C	0.89	17.33	13.41	90.49	6.83		
**Original Texture**			**Matched Texture**				Direct Binarization	Texture-based
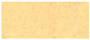			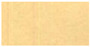				dSLR-C 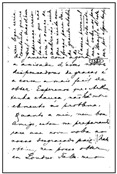	Howe-C 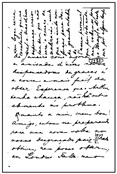
**Direct Binarization**								
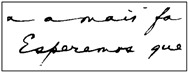								
**Texture-based binarization**								
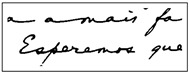								

**Table 8:** Results for image matching with image HW 05.

Binarization Results							Original Image	Matched Image
for the Original Image							HW 05	HW 06
#	**Algorithm**	**Kappa**	**PSNR**	**DRD**	**FM**	**Time**	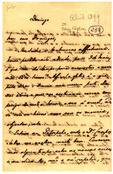	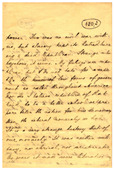
1	Jia-Shi-L	0.92	18.73	25.58	93.23	4.61		
1	Jia-Shi-R	0.92	18.57	25.51	92.91	4.61		
2	DocDLink-C	0.91	18.03	27.56	91.85	4.08		
3	Jia-Shi-B	0.90	17.53	32.94	91.23	4.50		
4	DocDLink-L	0.89	17.18	35.56	90.31	4.01		
5	DocDLink-B	0.88	16.58	41.27	88.96	3.98		
6	Lu-Su-B	0.86	15.85	48.36	87.59	14.78		
6	Lu-Su-C	0.86	15.71	51.69	87.25	14.14		
...	...	...	...	...	...	...		
11	Wolf-B	0.81	14.83	62.79	83.15	0.05		
**Original Texture**			**Matched Texture**				Direct Binarization	Texture-based
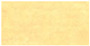			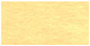				Jia-Shi-L 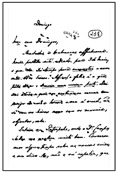	Wolf-B 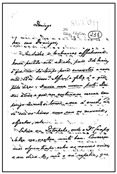
**Direct Binarization**								
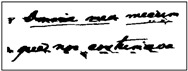								
**Texture-based binarization**								
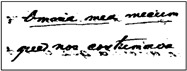								

**Table 9:** Results for image matching with image HW 06.

Binarization Results							Original Image	Matched Image
for the Original Image							HW 06	HW 05
#	**Algorithm**	**Kappa**	**PSNR**	**DRD**	**FM**	**Time**	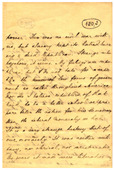	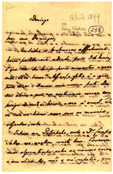
1	Wolf-B	0.93	20.74	8.98	93.32	0.05		
2	dSLR-B	0.92	20.24	10.66	92.72	0.01		
2	dSLR-G	0.92	20.01	11.42	92.44	0.01		
2	dSLR-L	0.92	19.92	11.15	92.10	0.01		
2	Intermodes-B	0.92	20.10	11.55	92.68	0.01		
2	Intermodes-C	0.92	19.78	12.28	92.09	0.01		
2	Intermodes-L	0.92	19.79	12.30	92.13	0.01		
2	Li-Tam-B	0.92	20.24	10.66	92.72	0.01		
...	...	...	...	...	...	...		
4	Jia-Shi-L	0.90	18.73	15.06	90.60	4.43		
**Original Texture**			**Matched Texture**				Direct Binarization	Texture-based
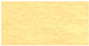			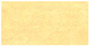				Wolf-B 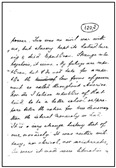	Jia-Shi-L 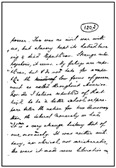
**Direct Binarization**								
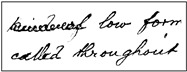								
**Texture-based binarization**								
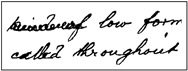								

**Table 10:** Results for image matching with image TW 01.

Binarization Results							Original Image	Matched Image
for the Original Image							TW 01	TW 07
#	**Algorithm**	**Kappa**	**PSNR**	**DRD**	**FM**	**Time**	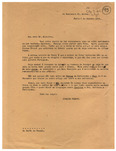	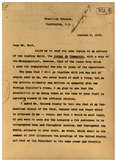
1	Li-Tam-C	0.94	23.84	4.20	94.18	0.02		
1	Li-Tam-G	0.94	23.94	4.16	94.30	0.02		
1	Li-Tam-L	0.94	23.53	4.56	93.81	0.01		
1	MO${}_{1}$-C	0.94	24.00	4.11	94.40	0.02		
1	MO${}_{1}$-G	0.94	24.09	4.09	94.50	0.02		
1	MO${}_{1}$-L	0.94	23.98	4.15	94.38	0.02		
2	Intermodes-B	0.93	23.29	5.30	93.35	0.02		
2	IsoData-B	0.93	23.29	5.30	93.35	0.02		
...	...	...	...	...	...	...		
6	Su-Lu-L	0.89	21.69	6.83	89.26	0.59		
**Original Texture**			**Matched Texture**				Direct Binarization	Texture-based
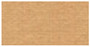			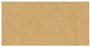				Li-Tam-L 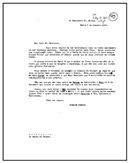	Su-Lu-L 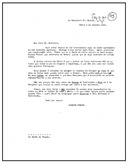
**Direct Binarization**								
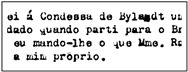								
**Texture-based binarization**								
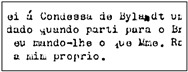								

**Table 11:** Results for image matching with image TW 02.

Binarization Results							Original Image	Matched Image
for the Original Image							TW 02	TW 11
#	**Algorithm**	**Kappa**	**PSNR**	**DRD**	**FM**	**Time**	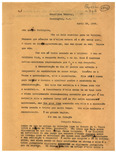	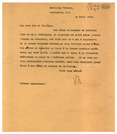
1	dSLR-C	0.96	23.20	3.95	96.28	0.02		
1	dSLR-G	0.96	23.07	4.06	96.09	0.02		
1	dSLR-L	0.96	23.18	3.89	96.19	0.02		
1	Intermodes-C	0.96	22.78	4.39	95.95	0.02		
1	Intermodes-G	0.96	22.77	4.46	95.93	0.02		
1	Intermodes-L	0.96	22.72	4.47	95.90	0.02		
1	Li-Tam-G	0.96	22.77	4.46	95.93	0.02		
1	Li-Tam-L	0.96	22.72	4.47	95.90	0.02		
...	...	...	...	...	...	...		
5	Otsu-G	0.92	19.78	9.04	92.31	0.01		
**Original Texture**			**Matched Texture**				Direct Binarization	Texture-based
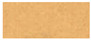			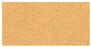				dSLR-C 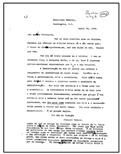	Otsu-G 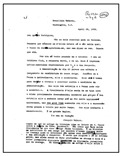
**Direct Binarization**								
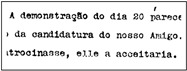								
**Texture-based binarization**								
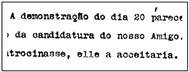								

**Table 12:** Results for image matching with image TW 03.

Binarization Results							Original Image	Matched Image
for the Original Image							TW 03	TW 10
#	**Algorithm**	**Kappa**	**PSNR**	**DRD**	**FM**	**Time**	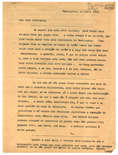	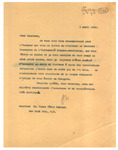
1	Minimum-C	0.97	24.80	4.82	96.99	0.02		
1	Nick-C	0.97	25.07	5.01	97.14	0.08		
1	Nick-G	0.97	24.62	5.53	96.81	0.07		
1	Nick-L	0.97	25.03	5.07	97.11	0.07		
1	Singh-C	0.97	25.33	4.87	97.33	0.12		
1	Singh-G	0.97	24.58	5.65	96.81	0.11		
1	Singh-L	0.97	25.06	5.13	97.16	0.12		
2	MinError-C	0.96	24.45	4.90	96.67	0.02		
2	MinError-L	0.96	24.19	5.14	96.43	0.02		
2	Nick-R	0.96	23.44	6.77	95.86	0.08		
**Original Texture**			**Matched Texture**				Direct Binarization	Texture-based
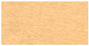			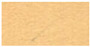				Minimum-C 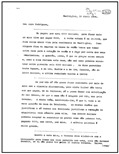	Minimum-C 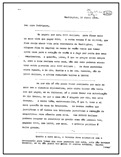
**Direct Binarization**								
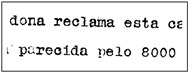								
**Texture-based binarization**								
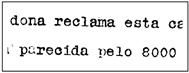								

## Data Availability

The results presented here made use of the IAPR (International Association on Pattern Recognition) DIB—Document Image Binarization dataset, available at: https://dib.cin.ufpe.br, last accessed on 24 August 2022.
